# Attention moderates the motion silencing effect for dynamic orientation changes in a discrimination task

**DOI:** 10.1167/jov.24.13.13

**Published:** 2024-12-20

**Authors:** Tabea-Maria Haase, Anina N. Rich, Iain D. Gilchrist, Christopher Kent

**Affiliations:** 1 School of Psychological Science, University of Bristol, Bristol, UK; 2 Macquarie University Performance and Expertise Research Centre and School of Psychological Sciences, Macquarie University, Sydney, Australia

**Keywords:** motion silencing effect, spatial attention, orientation change, discrimination task, Posner cueing, perceptual template model, crowding

## Abstract

Being able to detect changes in our visual environment reliably and quickly is important for many daily tasks. The motion silencing effect describes a decrease in the ability to detect feature changes for faster moving objects compared with stationary or slowly moving objects. One theory is that spatiotemporal receptive field properties in early vision might account for the silencing effect, suggesting that its origins are low-level visual processing. Here, we explore whether spatial attention can modulate motion silencing of orientation changes to gain greater understanding of the underlying mechanisms. In Experiment 1, we confirm that the motion silencing effect occurs for the discrimination of orientation changes. In Experiment 2, we use a Posner-style cueing paradigm to investigate whether manipulating covert attention modulates motion silencing for orientation. The results show a clear spatial cueing effect: Participants were able to discriminate orientation changes successfully at higher velocities when the cue was valid compared to neutral cues and performance was worst when the cue was invalid. These results show that motion silencing can be modulated by directing spatial attention toward a moving target and provides support for a role for higher level processes, such as attention, in motion silencing of orientation changes.

## Introduction

Our visual environment is rich in perceptual information, and one fundamental task for the visual system is to extract task relevant visual signals from irrelevant ones. External sources of noise may include shadows, occlusion, different luminance levels, and multiple objects that are stationary or in motion. Self-generated sources of noise include eye blinks, saccades, and body movement. Despite these perceptual challenges, our visual system can generate the mental representation of a stable visual world that allows us to perform goal-directed behaviors. Occasionally, however, this perceptual system fails, and investigating these errors can provide insights into how specific aspects of the visual system work. Such failures are often demonstrated through visual illusions—for example, motion-induced blindness. In this illusion, stationary objects in the periphery disappear and then reappear due to a moving (e.g., rotating) background (Bonneh, Cooperman, & Sagi, 2001). Our ability to reliably detect the presence and absence of visual targets and feature changes is often also crucial for performing a task—for example, detecting a traffic light changing color from red to green. Although we usually do this well, we can fail to notice significant changes under certain conditions, an obvious example being change blindness (e.g., Rensink, O'Regan, & Clark, 1997).

The motion silencing effect (Suchow & Alvarez, 2011) is another example of a failure to detect an otherwise obvious feature change, in this case due to the feature change being presented in a dynamic display. A similar effect, where a color switch from red to green is only reliably detected when the switch changes the summary statistics of the dynamic display, was documented at around the same time (Saiki & Holcombe, 2012). The implication of the motion silencing effect is that humans may fail to detect dynamic feature changes in more real-world dynamic environments, for example, when driving. It is therefore important to further understand the underlying mechanisms of this perceptual failure.

In the original demonstration of the motion silencing effect, Suchow and Alvarez (2011) showed that hue changes in a field of multicolored dots were noticed less when the display rotated compared with when it was stationary. They also showed this effect for size, luminance, and shape changes, highlighting the pervasive nature of this illusion and that a range of features at different levels of the visual processing hierarchy are affected. In their experiments, stimuli started as a stationary display, which would then rotate and change direction when it had rotated through 30°. The stationary and dynamic state reversed every 3 seconds, and participants had to adjust the rate of feature change in the stationary display to match the rate of change in the dynamic display. The key factor was that both rates of change started out the same and thus any difference between them should be due to the silencing effect. Suchow and Alvarez (2011) calculated a silencing factor based on this task by taking the ratio of the veridical to perceived rate of change as an indication of the strength of silencing. As the rotation of the display increased, so did the silencing factor, demonstrating a systematic relationship between rotation speed and the ability to perceive feature changes.

Suchow and Alvarez (2011) tested their illusion to determine whether or not the participants were able to perceive the new states of the changing features, which they termed “freezing,” or if participants perceived the new states accurately but were unaware of the small changes that led to the new states, known as “implicit updating.” The authors found that the latter contributed to silencing, as participants were able to detect large reversions in hue but had difficulty detecting smaller reversions in hue. Additionally, the authors tested whether retinal motion or spatial motion caused silencing. They created four variations of the initial silencing display that would cause spatial and retinal motion, retinal motion only, spatial motion only, or no motion at all. When comparing the silencing factors, the spatial and retinal motion variant and retinal motion alone produced the highest silencing factors. The authors concluded that retinal motion drives silencing over and above spatial motion.

Since the original motion silencing publication, various studies have further investigated this effect (Choi, Bovik, & Cormack, 2014; Choi, Bovik, & Cormack, 2016; Kavšek, 2013; Peirce, 2013; Turi & Burr, 2013). Peirce (2013) demonstrated that any salient visual change can cause silencing; for example, strong coherent color changes can silence the perception of motion (the inverse of the original silencing effect). Peirce also determined if coherence was a necessary part of silencing by assigning each dot stimulus a random speed and up- or downward trajectory, thereby ensuring that the global motion was not coherent. This still resulted in a silencing effect, with a significant perceived reduction in the rate of change compared to its true value. Therefore, neither coherence nor global motion seems necessary to generate silencing.

Choi et al., 2014; Choi et al. 2016 suggested that the motion silencing effect could be explained by the spatiotemporal response profiles of low-level receptive fields. The authors studied how silencing of luminance changes, or “flicker,” is affected by manipulations of dot spacing, flicker frequency, rotational speed, and eccentricity using human psychophysical and modeling-based approaches. They found that the degree of silencing is a combination of all of the latter properties with eccentricity, decreased dot spacing, flicker frequency, and increased rotational speed resulting in stronger silencing (Choi et al., 2014; Choi et al., 2016). The spectral analyses conducted on their stimuli and subsequent receptive field models revealed that, with increasing eccentricity, the response to the flicker and motion signals decreased. This lack of a large response reduced the population level response, leading to poorer flicker detection performance and thus silencing (Choi et al., 2016).

In contrast to this low-level receptive-field–based explanation of the motion silencing effect, Turi and Burr (2013) suggested that both crowding and global motion are responsible for the silencing. In their view, global motion effectively absorbs the information from the local feature changes, preventing the local “transients” from being detected. Crowding, a visual phenomenon where we cannot identify a target when it is located peripherally in a cluttered display (Bouma, 1970), may in turn blend the local features together, so that perceptual isolation of one feature to determine whether or not it is changing becomes impossible (Turi & Burr, 2013).

To investigate the effect of crowding on the motion silencing effect, Turi and Burr (2013) used a field of dots in three concentric rings with different radii, ranging from 6° to 10°. The dots in the middle annulus changed size in one of two intervals, and participants had to identify which interval showed the size change. The authors found that silencing was greater for displays that were more crowded. Their results for dot spacing aligned with Bouma's law (Bouma, 1970), as the critical spacing was around half the eccentricity. Turi and Burr (2013) suggested that crowding did not apply to stationary displays in the same way, as the transients of the feature changes were not masked by the global motion and therefore reached perceptual awareness. These experiments show that both inter-dot spacing and global motion have a role in silencing. Although Peirce (2013) showed that global motion is not necessary to produce silencing, the author acknowledged that global motion may better generate silencing compared to other salient visual changes.

Directing attention with an exogenous pre-cue decreases the amount of crowding (Yeshurun & Rashal, 2010). This may be because directing attention improves the observer's chance of localizing the change and integrating information over an appropriately sized integration field (Pelli, Palomares, & Majaj, 2004). We can use the perceptual template model (PTM) (Lu & Dosher, 1998; Lu & Dosher, 2008) to explain how spatial attention interacts with crowding to produce silencing. The PTM model has three key components: a template representing the target, a decision process, and sources of noise related to neural inefficiencies associated with the processing of the stimulus (Lu & Dosher, 1998). The signal is processed first by a matching to the template and this is followed by a perceptual decision. Within this model, attention can enhance the signal, exclude distracting noise sources, or reduce internal noise during the processing of the relevant signal. Attention may thus improve the detection or discrimination of feature changes during motion silencing either via the mechanism described in the PTM or simply through a reduction of crowding effects, or both.

Previously, some authors have noted that the silencing illusion is reduced when tracking one of the changing stimuli covertly (Saiki & Holcombe, 2012; Shioiri, Ogawa, Yaguchi, & Cavanagh, 2015; Suchow & Alvarez, 2017), but this does not completely abolish the effect, unlike some change blindness demonstrations (Burr, 2011). For example, Saiki and Holcombe (2012) showed that change is well detected when the proportion of color changes (summary statistics) is altered. In addition, in a stimulus display containing many dots, they demonstrated that participants are more sensitive to dot size changes when paying attention to a specific motion direction or color. Shioiri et al. (2015) further investigated the effect of attention on flickering target disks. In their change detection task, six disks surrounded fixation, and participants had to detect the presence or absence of flicker in the disks. A target disk could be pre-cued in comparative size to other disks, and participants were asked to monitor the target disk specifically when cued. Flicker sensitivity was higher compared to cued targets compared to non-cued targets, especially when the disks were rotating around fixation. Our experiments add to this existing literature by testing a novel feature change (orientation) for motion silencing in a change discrimination (not detection) task, directly manipulating spatial attention using Posner cueing, and using eye tracking consistently to ensure covert attention deployment, incorporating the results with the PTM and crowding.

The aims of this study were to test whether orientation change is also subject to the motion silencing effect and then to investigate if the motion silencing effect for orientation changes is modulated by spatial attention. If the latter is the case, then this suggests that any theoretical model of silencing (Choi et al. 2014; Choi et al., 2016) should take higher level cognitive modulation into account, with one explanation being that attention modulates the crowding component of the motion silencing effect proposed by Turi and others (e.g., Turi & Burr, 2013).

In Experiment 1, we established the presence of a silencing effect with dynamic orientation changes. Participants discriminated the direction of orientation change in objects. Consistent with the established motion silencing effect, we expected poorer discrimination performance with increasing rotational speed of the display. In Experiment 2, we manipulated spatial attention to investigate if attention decreases the silencing effect. Using the same stimuli as Experiment 1, we introduce a Posner-style cueing paradigm (Posner, 1980; Posner, Nissen, & Ogden, 1978) to direct covert spatial endogenous attention. We manipulated endogenous attention by using a line cue that extended from fixation toward one of the objects. We used three different cue types: a valid cue that directed attention toward an object with an orientation change on 75% of trials; an invalid cue that directed attention toward an object that was not changing orientation on 12.5% of trials; and a neutral cue that did not direct spatial attention to a particular object on the remaining 12.5% of trials. We expected the valid cue to improve discrimination performance and thereby show a reduced silencing effect. We expected the opposite pattern for the invalid cue type and for the neutral cue type to sit in between the other cue types as a baseline.

## Experiment 1

Experiment 1 investigated whether the motion silencing effect occurs for the discrimination of orientation changes when comparing static and dynamic displays. The experiment, hypotheses and analysis were pre-registered (https://osf.io/nkg29).

### Method

#### Participants

Twenty-two participants (17 females, 5 males; mean age, 21.9 years; range, 18–33) participated in this study and were reimbursed for the session with a £10 gift voucher or course credit. Ethical approval was gained from the University of Bristol's Faculty of Life Sciences Research Ethics Committee, and participants gave written informed consent.

#### Design

This fully within-subject study used a two (orientation change direction, clockwise or counterclockwise, at 120°/s) × two (annulus rotation direction, clockwise or counterclockwise) × nine (rotational speed: 0°, 12°, 24°, 36°, 48°, 60°, 72°, 84°, 96°; angular degrees per second) factorial design, where conditions were randomized within blocks. The dependent variables were response accuracy, correct and incorrect response times (RTs).

#### Materials

##### Apparatus

Stimuli were displayed on a 24-inch VIEWPixx3D Lite Monitor (VPixx Technologies, Saint-Bruno, QC, Canada), with a refresh rate of 120 Hz and a resolution of 1920 × 1080 pixels. Each participant sat 57 cm away from the monitor with their head stabilized by a chin and forehead rest. An EyeLink 1000 Tower Mount eye tracker (SR Research, Ottawa, ON, Canada) was used to monitor eye movements throughout the blocks at a sampling rate of 1000 Hz, and a USB keyboard was used to collect responses. Experiment control code was written in MATLAB R2019a (MathWorks, Natick, MA) using Psychtoolbox-3 (Brainard, 1997; Kleiner, Brainard, & Pelli, 2007; Pelli, 1997).

##### Stimuli

**Figure 1.**
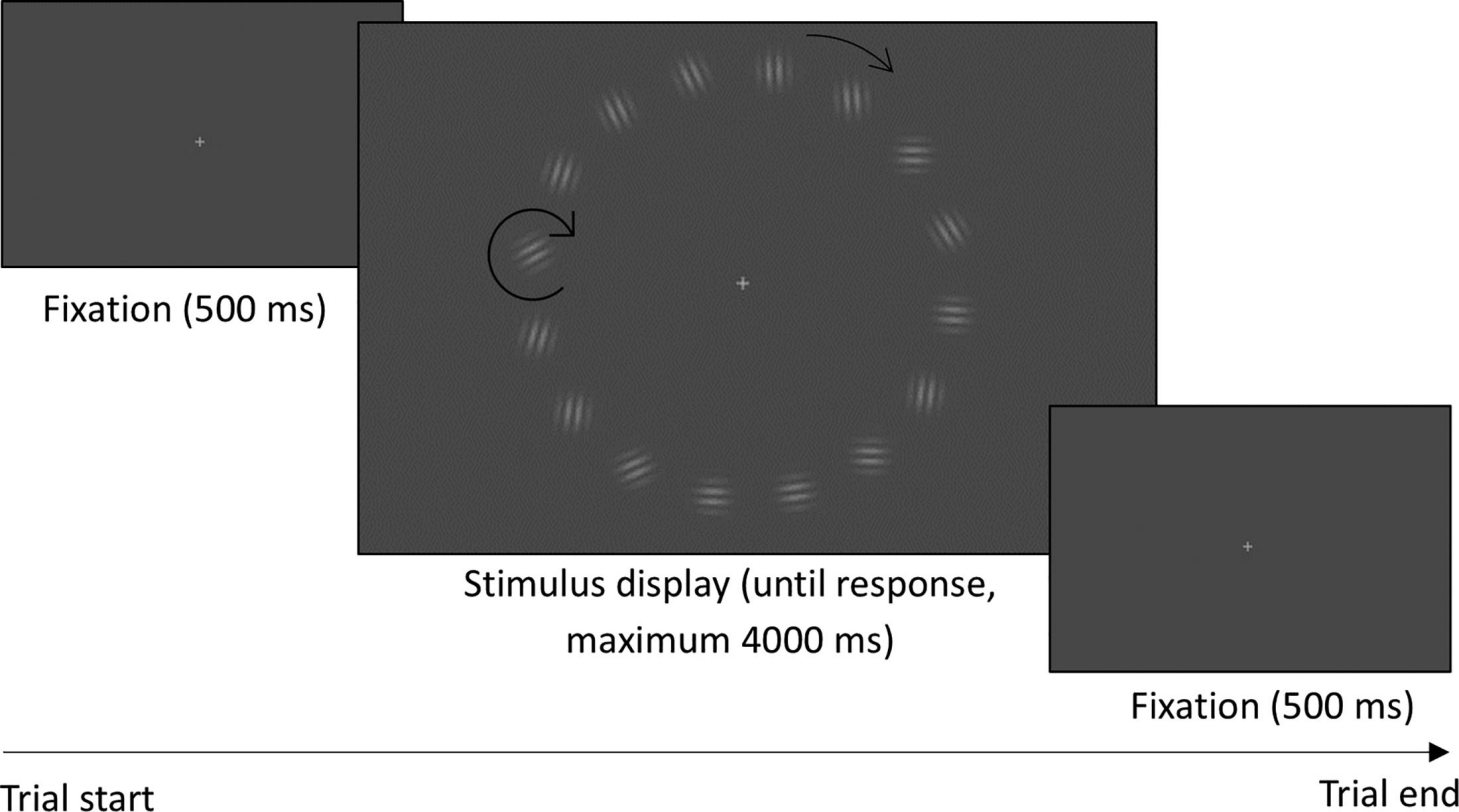
Example trial sequence for Experiment 1. The arrows represent example global and local rotation directions; in this trial, the annulus rotated clockwise and half of the individual Gabors rotated clockwise. The other half did not change orientation. The size and hue of the panels are exaggerated for clarity.

Sixteen Gabor patches (sinusoidal gratings within a Gaussian envelope) with close to 100% contrast, 5 cycles per degree spatial frequency, and a sigma of 0.40° visual angle were presented on a mid-gray background at an eccentricity (radius) of 5.58° visual angle in an annulus around fixation. Eight Gabor patches changed orientation dynamically and the other eight Gabor patches were static and did not change orientation. The Gabor patches that changed orientation did so continuously from trial onset, and all stimuli were presented with a random starting orientation on each trial. The location of changing and non-changing Gabors was random. The annulus rotated at one of the rotational velocities and directions (detailed above) per trial. The white fixation cross, 0.34° of visual angle in height and width, was located at the center of the screen. The background was mid-gray throughout the experiment. See Figure 1 for examples of the stimulus display. Stimuli were only presented when participants had been fixating centrally for at least 500 ms. An invisible window, 6.04° visual angle in height and width, was centered around fixation as the acceptable fixation area.

#### Procedure

The experiment was conducted in a quiet, darkened, windowless laboratory. A method of constant stimuli was used, where each type of trial was prespecified and only the presentation order was randomized. Each trial followed the same display sequence (see Figure 1). The eye tracker tracked the left pupil and was calibrated and validated using nine-point calibration before the start of each block and within each block after every 40 trials. Participants had a brief break every 20 trials, with instructions asking them to remain in position, rest their eyes, and return to the task when ready.

Participants were given oral and on-screen instructions prior to the start of the experiment and were given five practice trials, which were not included in the analysis. The fixation cross was presented for 500 ms, followed by the stimulus display until response for a maximum of 4000 ms. Throughout the stimulus display, the annulus rotated continuously either clockwise or counterclockwise at a constant annulus rotational speed. Note that, for the rotational speed of 0°/s the annulus did not rotate. At the same time, half of the Gabors changed orientation, all either clockwise or counterclockwise. The other half did not change orientation. Participants had to discriminate the direction of orientation change in the Gabor patches and indicate their response via a keyboard button press (right arrow key for clockwise and left arrow key for counterclockwise). When a response was given or 4000 ms was reached, the next trial started with the fixation display. The display sequence was triggered in a gaze-contingent manner, as participants’ fixations had to be inside the fixation window for 500 ms before the stimulus display was presented. If fixations were outside this window, the trial waited until the gaze had returned within the window for 500 ms. We also monitored eye movements throughout the stimulus display and excluded trials where participants were outside of our fixation window (see Results).

The practice trials followed the same format, except for the exact rotational speed and stimulus duration. The annulus rotated at a speed of 12°/s and the stimulus was displayed for a maximum of 10 seconds, giving participants ample time to familiarize themselves with the type of stimuli and the kind of change they were asked to discriminate. Each participant completed two blocks of 360 trials per block (total of 720 trials), which took approximately 1 hour. An animated demonstration of a practice and task trial can be found in the Supplementary Materials.

### Results

Data were analyzed using custom-written scripts in MATLAB R2023a (MathWorks), Excel (Microsoft, Redmond, WA), and JASP (Love et al., 2019); EyeLink edf files were converted using the Edf2Mat MATLAB Toolbox designed and developed by Adrian Etter and Marc Biedermann at the University of Zurich. Each participant's eye-tracking data were analyzed to determine their gaze location throughout the stimulus display to check fixations while the Gabors were displayed. Whenever fixations were outside the fixation window, the trial was indexed as a poor fixation trial. If more than 30% of trials were indexed as poor fixation trials, that participant's data were excluded. This was necessary because motion silencing only occurs in the visual periphery (Choi et al., 2016). A summary of the fixation data for each participant can be found in the Supplementary Materials. We excluded two participants who failed to meet the 30% threshold and another participant because the eye-tracking datafile for the second block was corrupted; therefore, 19 datasets were included in the final analyses. Overall, the mean percentage of fixations outside the central window were 11% (*SD* = 7.8) in Block 1 and 8% (*SD* = 7.4) in Block 2.

Next, we excluded all trials for which no response was given, and only trials with a response were further analyzed. The percentage of no-response trials excluded per participant can be found in the Supplementary Materials. Overall, 2.9% (*SD* = 5.1) of trials on average were excluded across participants. Each participant's discrimination task performance (proportion correct) was calculated for each rotational speed level. These data were fitted with an inverted cumulative normal distribution function, where the mean (μ), standard deviation (σ), and lapse rate (*L*) were free parameters, and the guess rate was constrained to 0.5. The cumulative normal distribution used was scaled between 0.5 (chance performance) and 1 (perfect performance) to model the data as follows:

$$\begin{array}{ccc} & & \;\mathrm{Proportion}\;\mathrm{correct}=\;\left(0.5-L\right)\\ & & \;\times f\left(\mathrm{annulus}\;\mathrm{rotational}\;\mathrm{velocity}\;x,\;\mu ,\sigma \right)+0.5 \end{array}$$

where *f*(annulus rotational velocity *x*, μ, σ) is the cumulative normal distribution at annulus rotational speed *x* (Kingdom & Prins, 2016, p. 79):

$$\begin{array}{c} F_{N}\left(x,\mu ,\;\sigma \right)=\;\frac{\sigma }{\sqrt{2\pi }}\int_{-\infty }^{x}\exp\left(-\frac{\sigma^{2}{\left(x-\mu \right)}^{2}}{2}\right) \end{array}$$

We used Microsoft Excel to fit the curves using the built-in Solver function (Fylstra, Lasdon, Watson, & Waren, 1998). All of the final individual participant curve fit plots can be found in the Supplementary Materials, as well as an example Excel curve fitting sheet. The curve fit for one participant (P8 in the Supplementary Materials) was not a good representation of their data, as the participant maintained high performance throughout the speed levels, with a mean proportion correct of 0.96 (range, 0.89–0.99). As the participant did not reach a 75% performance level, the estimate for this threshold may be unreliable; therefore, in line with our preregistration, the data of this participant were excluded from further analyses.

**Figure 2.**
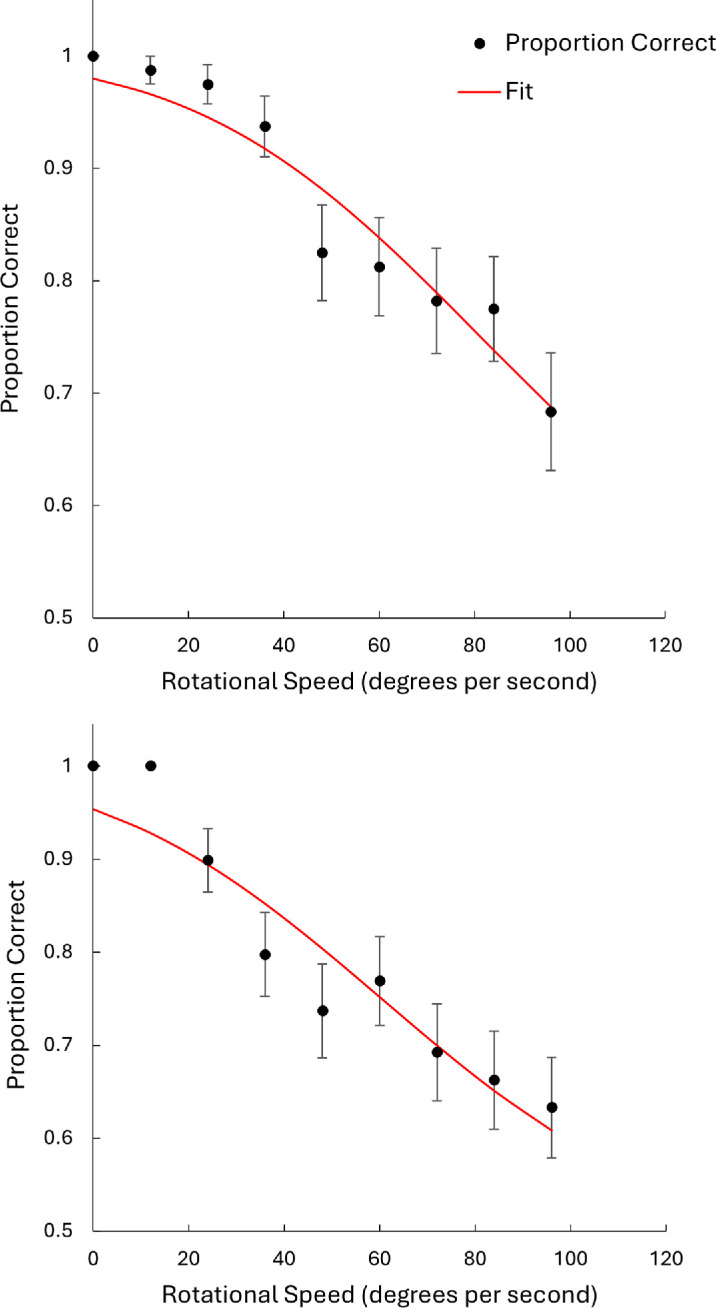
Representative psychometric curve fits for discrimination task performance. Error bars denote the standard error of proportion. Curve fits are from Participant 5 and Participant 12, respectively.

Figure 2 shows two representative curve fits. As can be seen, orientation discrimination performance deteriorated with increasing rotational speed, which is one of the key features of motion silencing (Suchow & Alvarez, 2011; Turi & Burr, 2013). The curve fits show that the inverted cumulative normal function was an appropriate model for our data, with average parameter estimates across participants being μ = 20.3° (range, 0°–6.14°), σ = 46.1 (range, 32.3–66.0), and *L* = 0.0004 (range, 0–0.008). As a goodness-of-fit estimate between the model and observed data, the mean *X*^2^(18) = 1.30 (*p* = 0.10; range, 0.18–3.60), suggesting that there was no reliable difference between the models and observed data.

**Figure 3.**
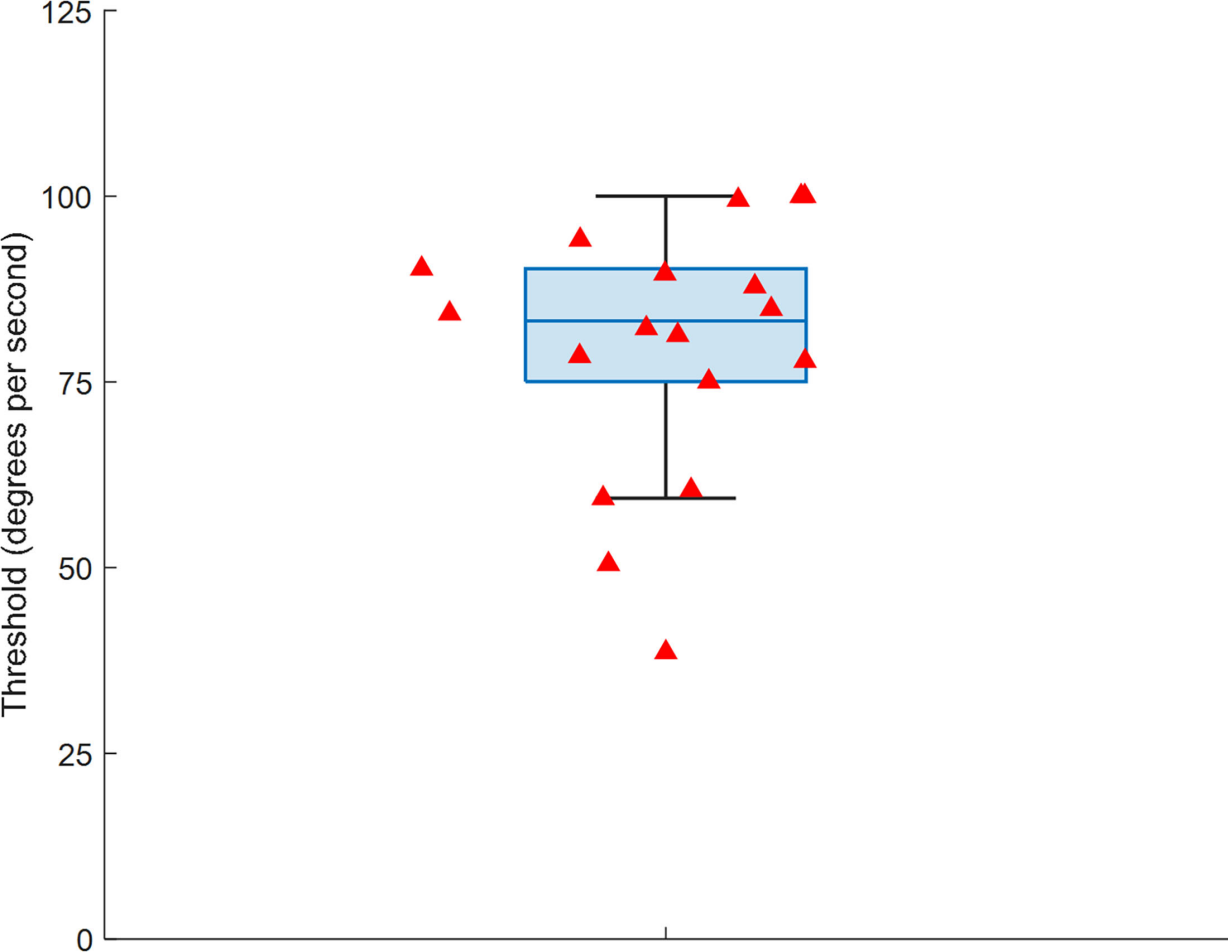
Box plot of participants’ 75% orientation discrimination thresholds. The red triangles represent individual participant 75% thresholds.

To further corroborate our finding of motion silencing, we tested whether the estimated standard deviation values from the curve fits differed from zero, which would demonstrate that accuracy in discriminating orientation change is a function of the rotational motion. A one-sample *t*-test was conducted on the standard deviation values derived from the model. There was a statistically significant difference between the standard deviation values (*M* = 46.1, *SD* = 9.6) and zero, *t*(17) = 20.3, *p* < 0.001. The effect size was large (Cohen's *d* = 4.79; 95% confidence interval [CI], 3.12–6.44). This result supports motion silencing in dynamic orientation discrimination. Figure 3 shows the individual participants’ 75% thresholds, taken as an index of motion silencing. The mean 75% performance threshold across participants was 79.7°/s (95% CI, 71.0–88.3). Finally, the mean RT across participants was 1513 ms (*SD* = 317), including both correct and incorrect trials. We did not find evidence for a speed–accuracy trade-off. We determined this by plotting the participant's mean RT versus their 75% threshold estimate. If participants had a fast RT and thus likely to make more errors, then this would be reflected in a lower threshold rotational speed and vice versa for slower RTs and higher accuracy. There was no clear pattern to this effect in the data, with a statistically non-significant correlation of *r*(16) = –0.04, *p* = 0.891.

### Discussion

Experiment 1 confirms that human observers show the motion silencing effect when discriminating the direction of orientation changes in dynamic Gabor patches, as we found a decrease in discrimination performance with increasing rotational speed. Participants were able to discriminate the direction of orientation changes with near ceiling accuracy when the annulus did not rotate and performed progressively worse with increasing levels of rotational speed. Even on an individual participant level, 75% performance thresholds clustered together, highlighting that most participants showed the silencing effect with orientation.

Silencing applies to feature changes that are processed throughout the visual hierarchy, ranging from low-level features such as luminance to higher level features such as hue (Suchow & Alvarez, 2011). The finding that silencing also occurs for orientation changes means that the low-level spatiotemporal energy model theory by Choi et al. (2014) can be extended from luminance flicker to orientation changes.

Having established that silencing takes place for our orientation discrimination task, we next tested whether attention plays a role in silencing for orientation changes. Although the effect of attention on motion silencing has been mentioned previously (Burr, 2011; Saiki & Holcombe, 2012; Shioiri et al., 2015; Suchow & Alvarez, 2017), it has not been investigated systematically for orientation changes. Attention underpins our ability to perform goal-directed behavior, by selecting information relevant to our goal from multiple potential sources of information (Carrasco, 2011). In terms of integrating this top–down process with visual processing, it has been shown that directing attention can improve performance on different visual tasks, such as detecting visual changes and thus reducing perceptual errors (Rensink, 2002). Additionally, directing attention to a spatial location covertly has been shown to enhance discriminability of low-level attributes such as contrast (Liu, Abrams, & Carrasco, 2009) and result in better resolution of spatial frequency (Abrams, Barbot, & Carrasco, 2010). More specific to the visual periphery and silencing, directing attention toward multiple targets that contain motion changes improves their detection (Vater, 2019).

Our second experiment manipulated covert endogenous attention using a Posner-style cueing paradigm (Posner, 1980; Posner et al., 1978) and a display in which only half the stimuli contained a feature (orientation) change. We hypothesized that a valid cue to a change would decrease silencing of orientation change discrimination relative to both a neutral cue condition and an invalid cue condition (where the invalid condition cued an unchanging Gabor).

## Experiment 2

Experiment 2 investigated how directing spatial attention would affect motion silencing of orientation changes using the motion silencing effect paradigm established in Experiment 1. The experiment, hypotheses, and analysis were pre-registered (https://osf.io/frx73). Only differences from Experiment 1 are described here.

### Method

#### Participants

Thirty-six participants (24 females, 12 males; mean age, 23.0 years; range, 18–38) participated in this study. Ethical approval was granted by the University of Bristol's Faculty of Life Sciences Research Ethics Committee, and participants gave written informed consent.

#### Design

This experiment used a fully within-subjects two (orientation change direction: clockwise or counterclockwise) × two (annulus rotation direction: clockwise or counterclockwise) × three (cue type: valid, invalid, or neutral) × eight (rotational speed: 12°, 24°, 36°, 48°, 60°, 72°, 84°, 96°; angular degrees per second) factorial design.

#### Materials

##### Stimuli

After the fixation cross, a placeholder display appeared, with dark gray circles placed around the locations where the Gabors would appear. These placeholder circles were 2.04° visual angle in size. This placeholder display was repeated for the subsequent cue display, with the addition of a line cue. For valid/invalid trials, the line cue (Vul & Rich, 2010) was a white line, 4.47° visual angle in size, extending from fixation toward the cued placeholder circle. For neutral trials, each placeholder circle had a small white cue line, halfway between fixation and its placeholder circle. These were 0.28° visual angle in size, and there were 16 of them so that they combined to be the same size as the valid/invalid cue line, maintaining the overall luminance change across display types.

#### Procedure

**Figure 4.**
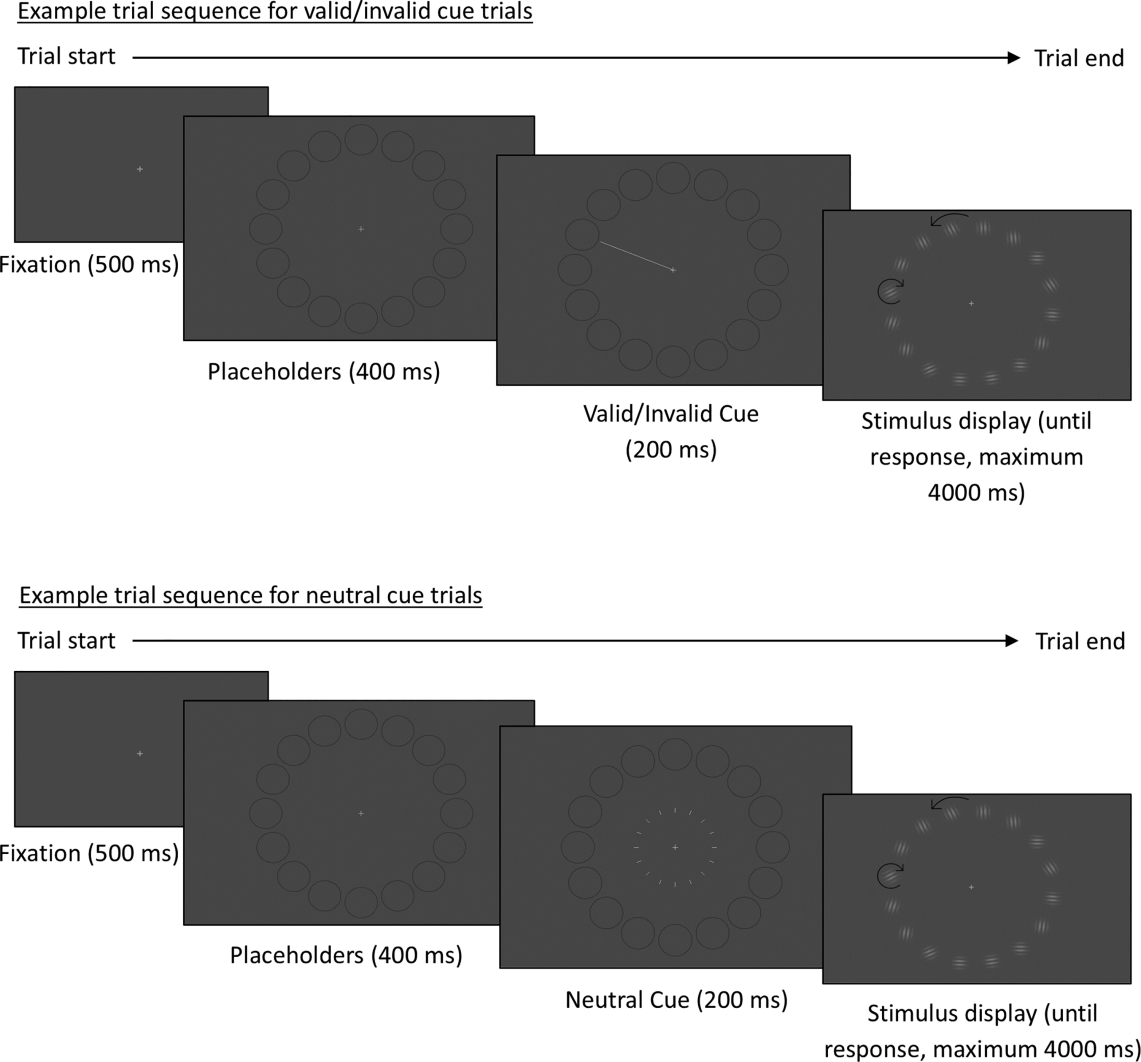
Example trial sequence for Experiment 2 showing the different cue types. The arrows represent example rotation directions; in these example trials, the annulus rotated counterclockwise and the Gabors rotated clockwise. The size and hue of the panels are exaggerated for clarity.

Participants were shown a short demonstration of the stimulus display prior to task onset to familiarize them with the stimulus display. Participants completed five blocks: a total of 500 trials composed of 300 valid trials, 100 invalid trials, and 100 neutral trials. Figure 4 shows an example trial sequence for Experiment 2. The trial started with a white fixation cross at the center of the screen for 500 ms, followed by the placeholder display for 400 ms. The cue display then appeared for 200 ms, followed by the stimulus display for 4000 ms, or until response, whichever occurred sooner.

This experiment used the psi-marginal adaptive staircase method (Kingdom & Prins, 2016; Prins, 2013) to estimate the 75% performance threshold as our parameter of interest. Our routine used uniform priors across the threshold, lapse, and slope parameters and fitted an inverted Weibull function to the data.

To ensure endogenous cueing, 75% of the cued trials were valid so that using the cue would help the participant complete the task. The valid cue pointed to a placeholder circle that contained a Gabor with an orientation change, which would make discrimination easier. The invalid cue pointed to a Gabor without an orientation change, thus requiring participants to reorient attention toward a Gabor with an orientation change, which would make discrimination more difficult, decrease the threshold estimates, and increase RTs. Threshold estimates must be computed over an equal number of trials for each condition to allow reliable comparison between the conditions. Therefore, only every third valid trial was used by the psi-adaptive marginal method to compute respective threshold estimates, but every trial was used by the psi-adaptive marginal method for the invalid and neutral trial types. Thus, 100 valid trials, 100 invalid trials, and 100 neutral trials were used by the staircases to compute respective 75% threshold estimates for each participant. The Palamedes toolbox (Prins & Kingdom, 2018) was used to implement three independent psi-marginal adaptive staircases running at the same time to derive thresholds related to discrimination performance at 75% (Talipski, Goodhew, & Edwards, 2022). Using these three staircases simultaneously also reduced the likelihood that participants would use a rule-based response system based on how the stimulus level changed on each trial, which can be a possibility when using single staircases (Kingdom & Prins, 2016, p. 131). Participants completed five blocks, resulting in a total of 500 trials composed of 300 valid trials, 100 invalid trials, and 100 neutral trials. Animated demos of the different cue type trials can be found in the Supplementary Materials.

### Results

All participants were within the fixation window at least 70% of the time (*M* = 90.6%, *SD* = 7.4%); therefore, no participants were excluded. We further checked the eye-tracking data across participants in response to a reviewer (thus departing from our pre-registration). Where a saccade was detected during the trial using standard EyeLink criteria, the horizontal and vertical mean landing positions of the subsequent fixation were 0.21° and 0.11°, and the standard deviations were 1.36° and 1.25°, respectively. This means that 75% of the saccades were within 1° of the central fixation point (0.9° vertically and 0.8° horizontally, respectively). Overall, these data suggest that, when a saccade was generated, the vast majority of the saccades were relatively small and around the fixation rather than toward the annulus. In addition, we checked the number of saccades for the different cue types. For valid trials, on 42.56% of trials no saccade was detected; where a saccade was detected, 51.94% of trials were inside and 5.50% of trials were outside the fixation window. For invalid trials, no saccade was detected in 38.22%; 56.11% were inside and 5.67% were outside the fixation window. For neutral trials, no saccade was detected in 42.22%; 52.00% were inside and 5.78% were outside the fixation window. This suggests that there were no large or systematic differences in eye movements between the different cue types, which in turn means it is unlikely that overt attention accounts for our results.

Trials where no response was given were included in the threshold estimation procedure and subsequent analyses. These non-response trials were excluded from RT data analysis. Across participants, 5.47% of trials were excluded due to a non-response in the RT analyses: 1.39% of trials were excluded in the valid cue, 1.89% in the invalid cue, and 2.19% in the neutral cue conditions.

#### Threshold estimation

**Figure 5.**
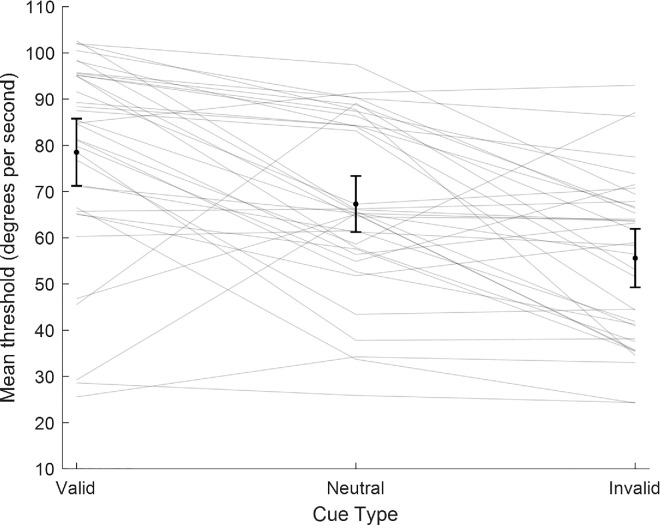
Mean threshold collapsed across participants by cue type. Individual data are shown as a line plot. Black dots show cue-type threshold means. Error bars denote (across participant) 95% confidence intervals.

A one-way repeated-measures analysis of variance (ANOVA) was conducted on the threshold estimates to compare the effect of cue type in valid, invalid, and neutral cueing conditions. There was a significant effect of cue type, *F*(2, 35) = 27.37, *p* < 0.001, η^2^ = 0.439. Two-tailed, paired-sample *t*-tests were then conducted to compare the cue validity conditions. As shown in Figure 5, there were differences in the thresholds for all condition comparisons. Valid cue thresholds (*M* = 78.5°, *SD* = 21.5°) were significantly higher than invalid thresholds (*M* = 55.6°, *SD* = 17.9°), *t*(35) = 6.98, *p* < 0.001, Cohen's *d* = 1.16 (95% CI, 0.73–1.58). Neutral cue thresholds (*M* = 67.3°, *SD* = 18.7°), *t*(35) = 3.54, *p* = 0.001, Cohen's *d* = 0.59 (95% CI, 0.23–0.94),^1^ and invalid cue thresholds (*M* = 55.6°, *SD* = 17.9°) were significantly lower than neutral cue thresholds (*M* = 67.3°, *SD* = 18.7°), *t*(35) = 4.15, *p* < 0.001, Cohen's *d* = 0.69 (95% CI, 0.32–1.05). This pattern of data suggests that directing spatial attention improved performance and modulates motion silencing in orientation change discrimination.

#### Response-time analysis

**Figure 6.**
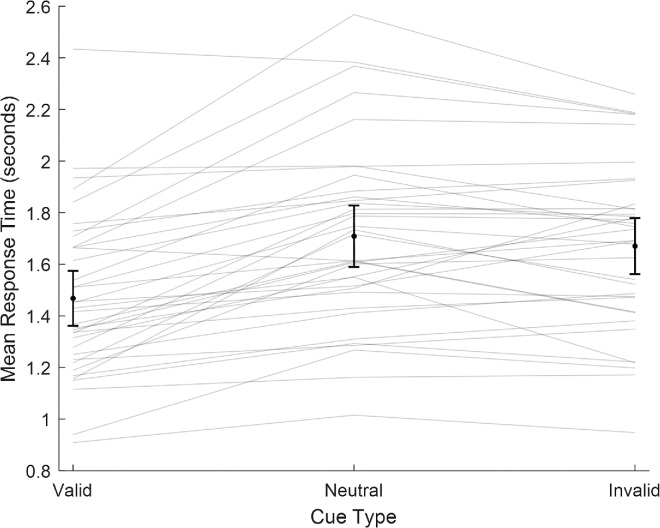
Mean RTs collapsed across participants by cue type. Individual data are shown as line a plot. Black dots show cue-type RTs. Error bars denote (across participant) 95% confidence intervals.

Although not the primary performance measure, the RT data analysis showed a trend similar to that of the threshold data. We expected the valid cue to improve performance by alerting the observer to a relevant location and feature change, thus reducing the RT necessary to discriminate orientation. We also expected the reverse pattern to occur for invalid cues, as they misdirected spatial attention and thus participants would have to reorient their attention to a relevant location before making their discrimination judgment, thereby increasing RTs. Figure 6 shows the mean RT per cue type for each participant.

A one-way repeated-measures ANOVA was conducted on RTs to compare the effect of cue type among valid, invalid, and neutral cueing conditions. There was a statistically significant effect of cue type, *F*(2, 35) = 36.1, *p* < 0.001. Two-tailed paired-sample *t*-tests were then conducted to test the effect of cue validity. As shown in Figure 6, there were differences in the RTs for two out of the three condition comparisons. Valid cue RTs (*M* = 1.47 s, *SD* = 0.315 s) were significantly lower than invalid cue RTs (*M* = 1.67 s, *SD* = 0.321 s), *t*(35) = 6.02, *p* < 0.001, Cohen's *d* = 1.00 (95% CI, 0.60–1.40). Valid cue RTs were also significantly lower than neutral cue RTs (*M* = 1.71 s, *SD* = 0.352 s), *t*(35) = 7.35, *p* < 0.001, Cohen's *d* = 1.23 (95% CI, 0.79–1.66). There were no reliable differences in the RTs for the neutral cue type (*M* = 1.71 s, *SD* = 0.352 s) and invalid cue type (*M* = 1.67 s, *SD* = 0.321 s), *t*(35) = 1.59, *p* = 0.121, Cohen's *d* = 0.27 (95% CI, 0.07–0.60).^2^

#### Non-response analysis

The psi-adaptive marginal method (Prins, 2013; Prins & Kingdom, 2009) used to compute the participants’ thresholds coded a non-response as incorrect. We checked the pattern of average discrimination threshold results depending on the number of non-responses participants had to ensure including non-responses did not impact our results. Only 5.47% of trials across participants were non-response trials. The results we found for the initial threshold analysis persisted and non-responses did not vary by cue type. Therefore, we conclude that it is unlikely that non-responses being coded as an error by the staircase had a confounding effect on our pattern of results (see Supplementary Materials for further details).

### Discussion

Experiment 2 tested the hypothesis that directing spatial attention covertly to a change would improve orientation discrimination performance and thus dampen the silencing effect. We observed a clear cueing effect, where participants showed improved discrimination and reaction time performance with a valid cue and worse discrimination performance with an invalid cue, supporting our hypotheses.

There are different underlying mechanisms that may explain how attention modulates the silencing effect. Some possible candidates include that directing spatial attention may improve the appearance of peripheral Gabors by increasing their contrast (Liu et al., 2009) or spatial frequency (Abrams et al., 2010), or attention may increase the spatial resolution of the visual field to which attention is directed (Carrasco & Barbot, 2015; Yeshurun & Carrasco, 1998). Alternatively, directing spatial attention with a cue may improve access to the orientation information contained in early visual processing areas by reducing spatial uncertainty. We discuss possible mechanisms further below.

## General discussion

We investigated whether the motion silencing effect (Suchow & Alvarez, 2011) occurs for orientation (Experiment 1) and whether it is modulated by spatial attention (Experiment 2). We found clear evidence that silencing occurs for orientation changes and that spatial attention reduces this effect.

In Experiment 1, we ran an orientation discrimination task to investigate the relationship between participants’ orientation discrimination performance and annulus rotation speed. Consistent with the established motion silencing effect, we found an inverse relationship, where an increase in rotation speed was linked to a decrease in discrimination performance. Participants were able to perform the discrimination task very well when the annulus did not rotate but progressively performed worse as the rotational speed increased. The inverse relationship between rotation speed and performance is a hallmark of the silencing effect and is well documented (Choi et al., 2014; Suchow & Alvarez, 2011; Turi & Burr, 2013). Silencing has been shown for other feature changes that are processed throughout the visual hierarchy, ranging from very low-level, such as luminance (Choi et al., 2014; Suchow & Alvarez, 2011) to higher level feature changes, such as hue or shape (Suchow & Alvarez, 2011). Here, we demonstrated that the motion silencing effect extends to orientation.

In Experiment 2, we investigated the effect of manipulating covert spatial attention (Burr, 2011; Posner, 1980; Posner et al., 1978; Saiki & Holcombe, 2012; Shioiri et al., 2015; Suchow & Alvarez, 2017) on silencing in orientation changes. The results showed that a valid cue improved discrimination performance and lowered RTs compared with an invalid cue, which misdirected attention toward a Gabor without an orientation change. There are multiple mechanisms by which spatial attention might improve task performance.

If we return to the framework of the perceptual template model (Lu & Dosher, 1998; Lu & Dosher, 2008), there are three main ways in which attention might be affecting perceptual processing throughout the model: stimulus enhancement, distractor exclusion, and internal noise reduction. Of these three, the only mechanism that directly modifies the perceptual template is distractor exclusion. For signal enhancement, attention influences the output of the perceptual template, and, for internal noise reduction, attention affects the impact of noise sources following the output of the perceptual template (Lu & Dosher, 1998).

Stimulus enhancement suggests that attention improves the output of the perceptual template at the attended region (Lu & Dosher, 1998). In our study, attending toward the area containing a Gabor with an orientation change may have increased the gain of the matching perceptual template output. However, this enhancement only benefits discrimination performance if external noise sources are not too high; otherwise, both the signal and noise are enhanced (Lu & Dosher, 1998). Distractor exclusion suggests that the perceptual template is narrowed by attention, which means that it is better tuned to respond to the signal (e.g., clockwise/counterclockwise orientation changes in our study) and responds less to any distractors (e.g., Gabors without an orientation change). This mechanism results in improved task performance if the stimulus display contains sufficient sources of external noise to be excluded, such as the high annulus speed trials in our experiment. Similarly, in Experiment 1, for trials where the annulus was stationary, external noise was low, so pre-attentive processes were able to detect the motion transients caused by the orientation changes, which caused the absence of silencing and high performance for those trials, in line with the conclusions by Turi and Burr (2013).

Finally, attention may also reduce the noise associated with neural processing inefficiencies, where a reduction in additive internal noise is defined as mathematically equivalent to signal enhancement. Attention may reduce the gain of multiplicative internal noise, and this would improve performance across levels of external noise, though especially in high external noise displays (Lu & Dosher, 1998).

Relatedly (Zhaoping, 2019; Zhaoping, 2024) proposed a framework that predicts that peripheral vision is more prone to visual illusions. In this framework, V1 generates a saliency map in which visual field locations with high bottom–up saliencies are highlighted by increased V1 activity to attract gaze shifts and attention. This motivates an attentional bottleneck right after V1, which means only a fraction of visual information is fed forward to be processed further. This bottleneck, by limiting information, causes information ambiguity, such that the Gabor patch rotation directions in our experiments would be ambiguous. Zhaoping (2019) proposed that such an ambiguity could be resolved for discrimination in central vision via top–down feedback to query for additional information using a process referred to as *analysis* *by* *synthesis*. This process determines the veridical input (the rotation direction, in our case) by having higher visual areas generate potential visual inputs for each rotation direction. These would-be inputs are then fed back to check whether they match the actual V1 signals. In addition, the framework proposes that peripheral vision, whose role is shifting attention rather than visual discrimination, lacks such a feedback query and is therefore more vulnerable to illusions and crowding caused by the ambiguity.

In our experiment, trials with higher rotations speeds could represent high external noise trials and vice versa for low rotation speed trials. In addition, at the tested eccentricity, peripheral vision may be of too low acuity to precisely localize the orientation changes when the annulus moves rapidly, thus introducing noise from the rotation, the lower acuity, and the decreased peak spatial frequency sensitivity we find in the visual periphery (Kerr, 1971; Kitterle, 1986). The strength of the local orientation signal was kept constant in our studies, because the rate of orientation change did not vary. As we derived a discrimination threshold across rotational velocities and did not specifically manipulate levels of stimulus noise beyond rotational velocity, it is difficult to pinpoint which one, or combination, of these three mechanisms is responsible for the overall beneficial effect of attention. Overall, the cue directed spatial attention and thus may have enhanced the signal or reduced some of the external noise inherent in the stimulus display, allowing participants to detect the direction of orientation change. Future work can build on this idea and determine the underlying mechanism of how the cue improved orientation discrimination.

The results of both experiments can be united with the findings from Choi et al. (2014), which show that the strength of the motion silencing effect is linked to stimulus velocity, the strength of feature change, and the spacing between items. Their low-level model of silencing predicts that silencing should occur with orientation changes, as with sufficient rotational velocity, the orientation changes may not be sampled quickly enough by the receptive fields to discriminate orientation change. We observed this behavioral pattern of results in Experiment 1 and therefore our results are in line with their previous predictions and findings.

The concept of receptive field perceptual templates can also be reconciled with this low-level spatiotemporal flicker detector model. For example, the simple filter based on V1 receptive field properties in the Choi et al. model is constrained in its ability to respond to luminance changes. However, when attention reduces external noise and enhances the signal, the filter may be able to respond to relevant information. It is possible that this is reflected in the finding of increased flicker frequency resulting in higher silencing thresholds (Choi et al., 2014), as increased flicker represents a stronger signal, similar to how attention may enhance a signal. We need more work to elucidate the relationship among attention, PTM candidate mechanisms, and their effect on the model by Choi et al. (2014), for both orientation and luminance changes and for different levels of external noise. For now, attention evidently affects motion silencing for orientation changes and thus perceptual models must account for both bottom–up and top–down influences. A purely low-level model that excludes attention effects will not capture the full underlying processing mechanism.

In addition, we need to consider the idea that close spacing between items increases motion silencing, as shown by Turi and Burr (2013), who suggested that silencing is due to the level of crowding in the stimulus display and global motion subsuming local changes. However, as shown in our first experiment, participants were able to discriminate changes well when stationary, so if crowding is mainly responsible for silencing then we should also observe it for stationary stimulus displays. Turi and Burr (2013) did suggest that, when the display is stationary, the feature change transients are able to stand out and signify perceptual change. However, when the display starts to move, these feature change transients are less obvious, due to the strong global motion signal. The extent of this masking by global motion depends on how fast the annulus rotates. It is not clear which underlying mechanisms would have to change or process information differently to allow the continuum of perceiving change to not perceiving change with increased rotational velocity, as it is not an all-or-nothing phenomenon. Additionally, crowding itself does not describe a mechanism; rather, it represents the outcome of a visual process (Harrison & Bex, 2017). Therefore, although crowding is a contributing factor, silencing does not seem fully explained by crowding and global motion.

The improved performance due to directing spatial attention with a pre-cue may alleviate whatever contribution crowding makes to motion silencing (Carrasco, 2011; Gong, Liu, Liu, Huangfu, & Geng, 2023; Turi & Burr, 2013; Yeshurun & Rashal, 2010). To discriminate orientation change, our perceptual system combines the output of numerous detectors from relevant spatial locations into an integration field (Carrasco, 2011; Pelli et al., 2004). As receptive fields increase in size in the periphery (Dumoulin & Wandell, 2008; Wilson & Sherman, 1976), so does the integration field (Carrasco, 2011; Pelli et al., 2004). When the integration field is too large, crowding occurs, as non-target information is erroneously processed (Carrasco, 2011). Alternatively, the attention resolution model of crowding (He, Cavanagh, & Intriligator, 1996; Intriligator & Cavanagh, 2001) suggests that the resolution of attention is too coarse to accurately select the target and thus information about distractors is also processed. Using our pre-cue, we reduced spatial uncertainty and may have reduced the erroneously large integration field or boosted the attentional resolution, possibly via the mechanisms of the PTM (Gong et al., 2023; He et al., 1996; Intriligator & Cavanagh, 2001; Lu & Dosher, 1998; Pelli et al., 2004). Thus, throughout our display, crowding and external noise in the stimuli may have been reduced, allowing the change signal to be detected and processed more efficiently, resulting in dampened motion silencing and enhanced discrimination performance.

Although it is possible that other attentional manipulations, such as an exogenous peripheral cue, may affect motion silencing differently, previous research has shown similar performance benefits in other paradigms for such cues. For example, an exogenous cue can result in decreased contrast detection thresholds (Carrasco, 2011). Therefore, it is likely that exogenous cues would also dampen silencing. To better understand the exact mechanism of how attention reduces silencing, it may be interesting to investigate the effect of a post-cue on silencing (Sergent et al., 2013). Here, a line cue similar to the one used in our study could be presented to cue a specific Gabor after the stimulus display was shown. If the pattern of results is similar, then this suggests a further role for sensory or visual short-term memory in the silencing effect.

Previously, motion silencing has been shown using change detection (Choi et al., 2014) and the method of adjustment tasks (Suchow & Alvarez, 2011), two-alternative forced-choice paradigms (Turi & Burr, 2013), and point of subjective equality (Peirce, 2013) studies. Our experiments add to this literature and reveal that motion silencing also applies to orientation discrimination tasks. Future work can broaden the investigation to explore the effect of different noise levels in the stimuli, different cue validities, or the effect of mental load on sustaining the attentional modulation on silencing.

## Conclusions

We investigated the motion silencing effect for dynamic orientation changes using a discrimination task. We found that participants’ ability to discriminate orientation changes deteriorated as the rotational speed of our annulus increased—a demonstration of the silencing effect for orientation. We then manipulated covert endogenous attention using a Posner-style cueing paradigm and demonstrated that a valid pre-cue to a location containing a change improved participants’ discrimination task performance thresholds relative to cues that shifted attention to a non-change location (invalid cue). This effect of attention on silencing of orientation changes is discussed with reference to the perceptual template model of attention and the possible role of crowding in the motion silencing effect. We suggest that a theoretical model of the silencing effect should take top–down and bottom–up effects into account, whereby the model by Choi et al. (2014) may benefit from the inclusion of a mechanism that represents attention effects on silencing.

## Supplementary Material

Supplement 1

## Footnotes

1 For the valid to neutral cue comparison, the Shapiro–Wilk test of normality suggested that the distribution of these data departed from normality (*W* = 0.929, *p* = 0.023). Therefore, the Wilcoxon signed-rank test was computed for the latter comparison and indicated a similar outcome: valid cue thresholds (*M* = 78.5°, *SD* = 21.5°) higher than neutral cue thresholds (*M* = 67.3°, *SD* = 18.7°, *T* = 550, *z* = 3.41, *p* < 0.001).

2 For the valid to neutral cue comparison, the Shapiro–Wilk test of normality suggested that the distribution of these data departed significantly from normality (*W* = 0.932, *p* = 0.028). Therefore, the Wilcoxon signed-rank test was also used for the latter comparison and indicated a similar outcome: valid cue type RT (*M* = 1.47 s, *SD* = 0.315 s) lower than neutral cue type RT (*M* = 1.71 s, *SD* = 0.352 s), *T* = 9.00, *z* = 5.09, *p* < 0.001.
