# Supplementary material for: Attention moderates the motion silencing effect for dynamic orientation changes in a discrimination task
Source: J Vis. 2024 Dec 20;24(13):13. doi: 10.1167/jov.24.13.13 (PMC11684489; doi:10.1167/jov.24.13.13)
Supplement: Supplement 1 [file jovi-24-13-13_s001.zip › Experiment_2_Supplementary_Information/Experiment_2_Non-responses_further_information.docx]

**Experiment 2**

Further information for non-response analysis

The Psi-adaptive marginal method (Prins, 2013; Prins & Kingdom, 2009) used to compute the participants’ thresholds coded a withheld response as incorrect. This became evident after the experiment had been conducted and data collection finished, which is why the following was not included in the pre-registration.

We checked the pattern of average discrimination threshold results depending on how many withheld responses participants had, ranging from none to a maximum of 29 (see Figure 1). The results we found for the initial threshold analysis persisted, with the trend of a lower discrimination threshold, and thus improved discrimination performance, for a valid cue in comparison to a higher discrimination threshold for an invalid cue. We also checked that no cue type had an undue number of withheld responses for each stimulus level (see Figure 2) and found that withheld responses were mostly evenly distributed. Therefore, we conclude that it is unlikely that withheld responses being coded as an error by the staircase had a confounding effect on our pattern of results.

Figure 1

*Scatter Plot showing Average Threshold by Cue Type for Categories of Number of Withheld Response Trials*

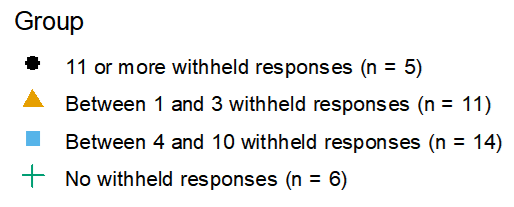


*Note. n* = number of participants in that group.

Figure 2

*Histogram of the Frequency of Withheld Responses for each Annulus Speed, by Cue Type*
