# Supplementary figures and images for: Attention moderates the motion silencing effect for dynamic orientation changes in a discrimination task
Source: J Vis. 2024 Dec 20;24(13):13. doi: 10.1167/jov.24.13.13 (PMC11684489; doi:10.1167/jov.24.13.13)

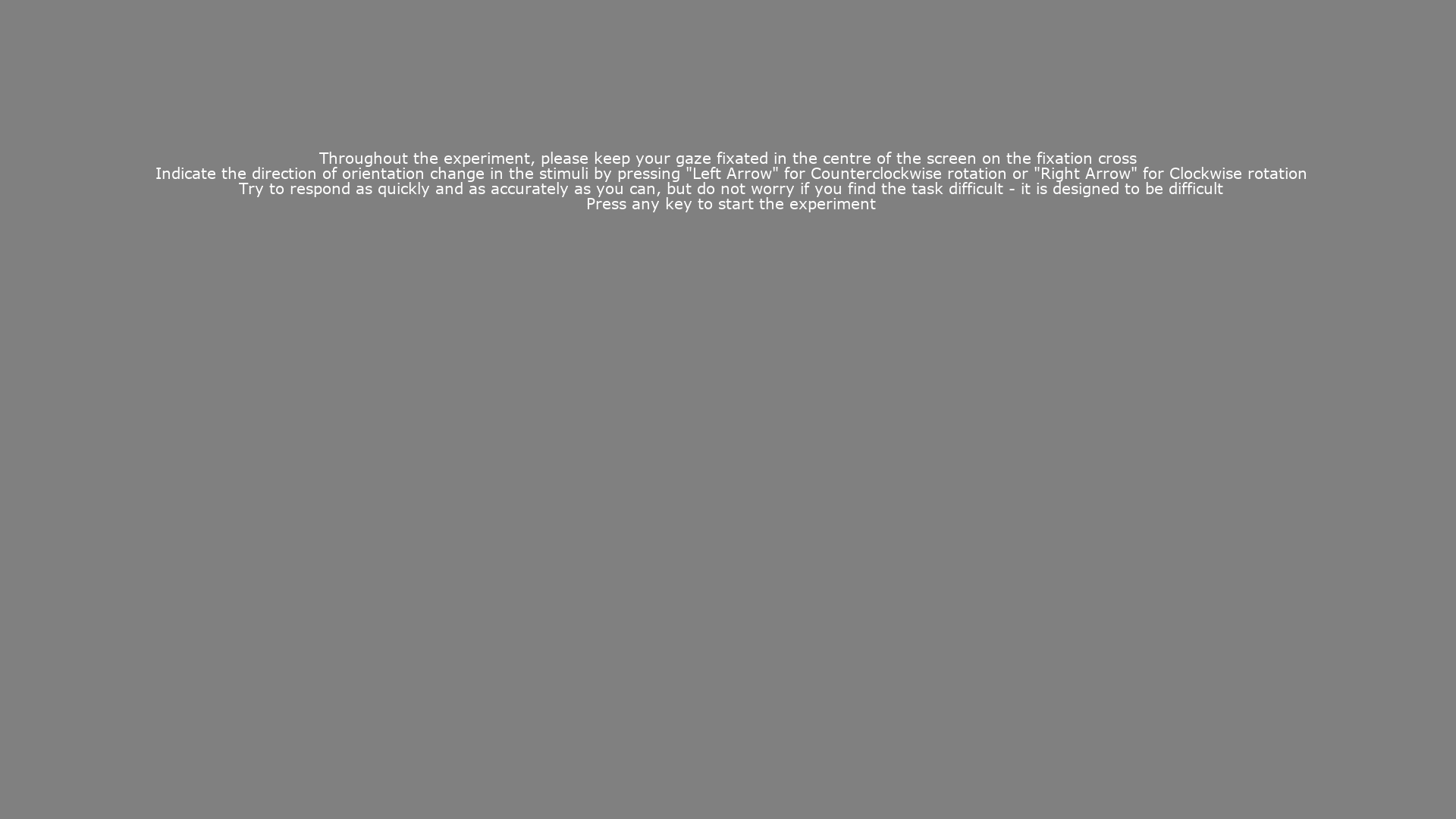

Supplement: Supplement 1 [file jovi-24-13-13_s001.zip › Experiment_1_Supplementary_Information/Experiment_1_Task.gif]

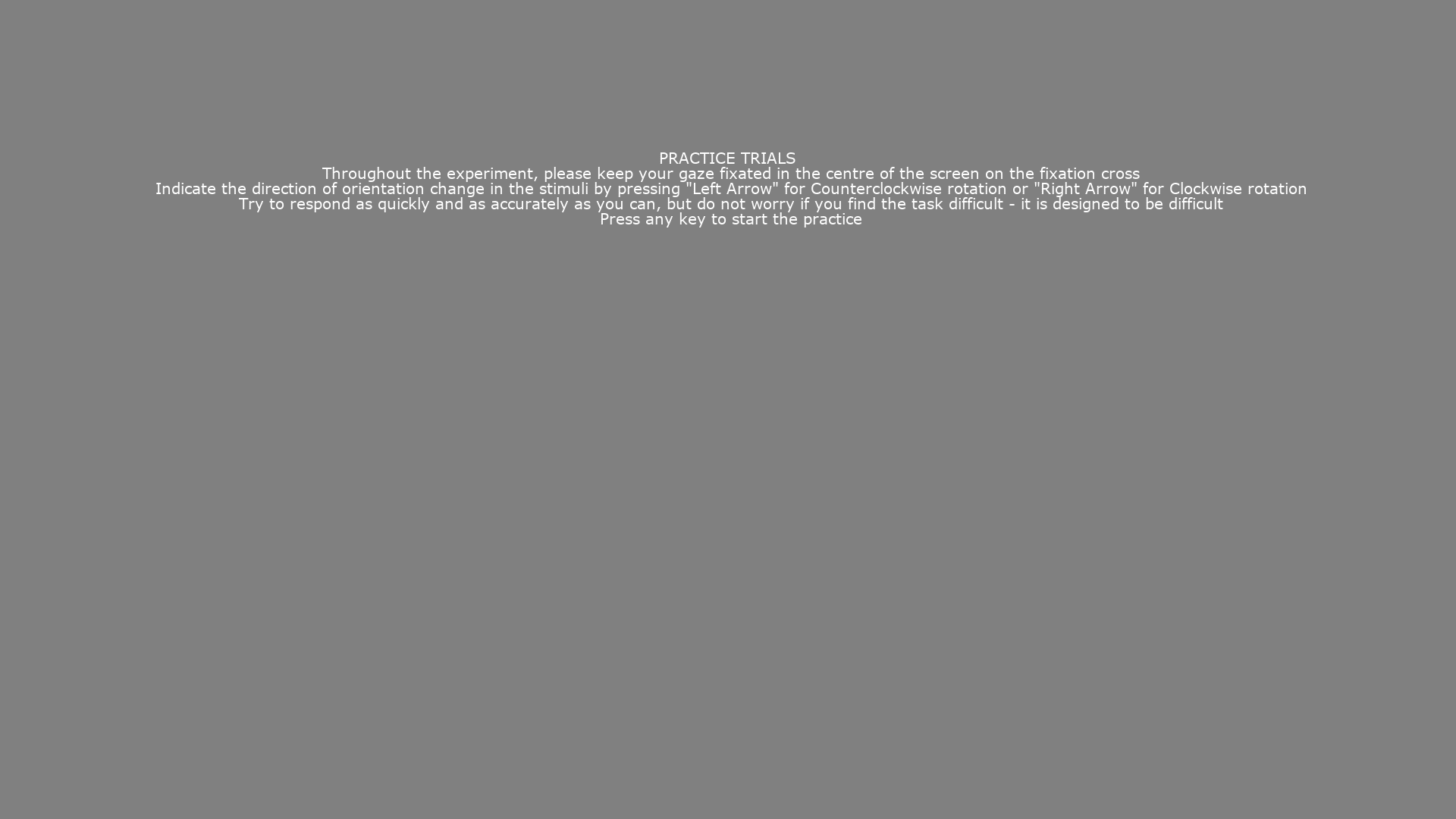

Supplement: Supplement 1 [file jovi-24-13-13_s001.zip › Experiment_1_Supplementary_Information/Experiment_1_Practice.gif]

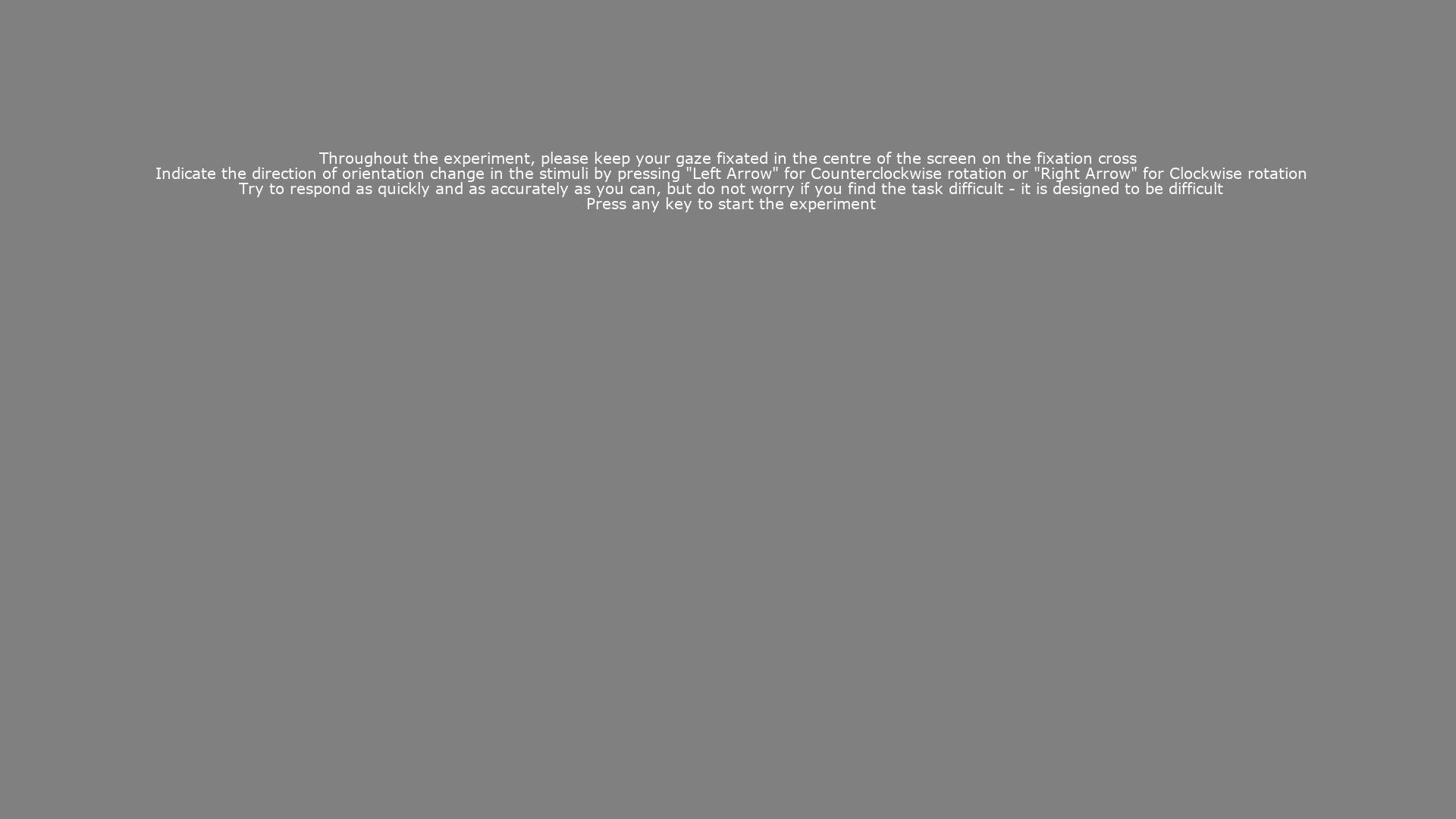

Supplement: Supplement 1 [file jovi-24-13-13_s001.zip › Experiment_2_Supplementary_Information/Experiment_2_Valid_Cue_Type.gif]

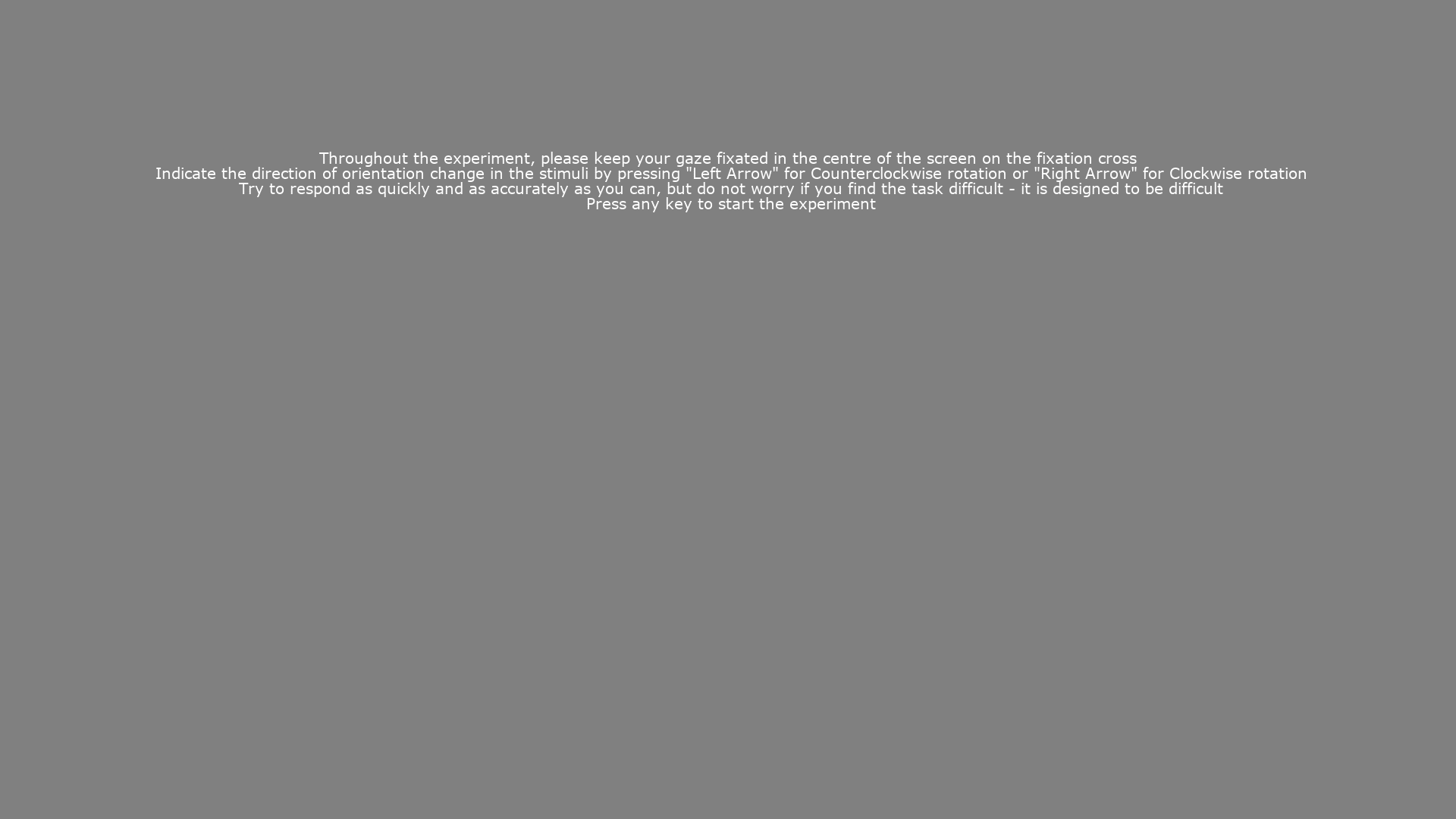

Supplement: Supplement 1 [file jovi-24-13-13_s001.zip › Experiment_2_Supplementary_Information/Experiment_2_Invalid_Cue_Type.gif]

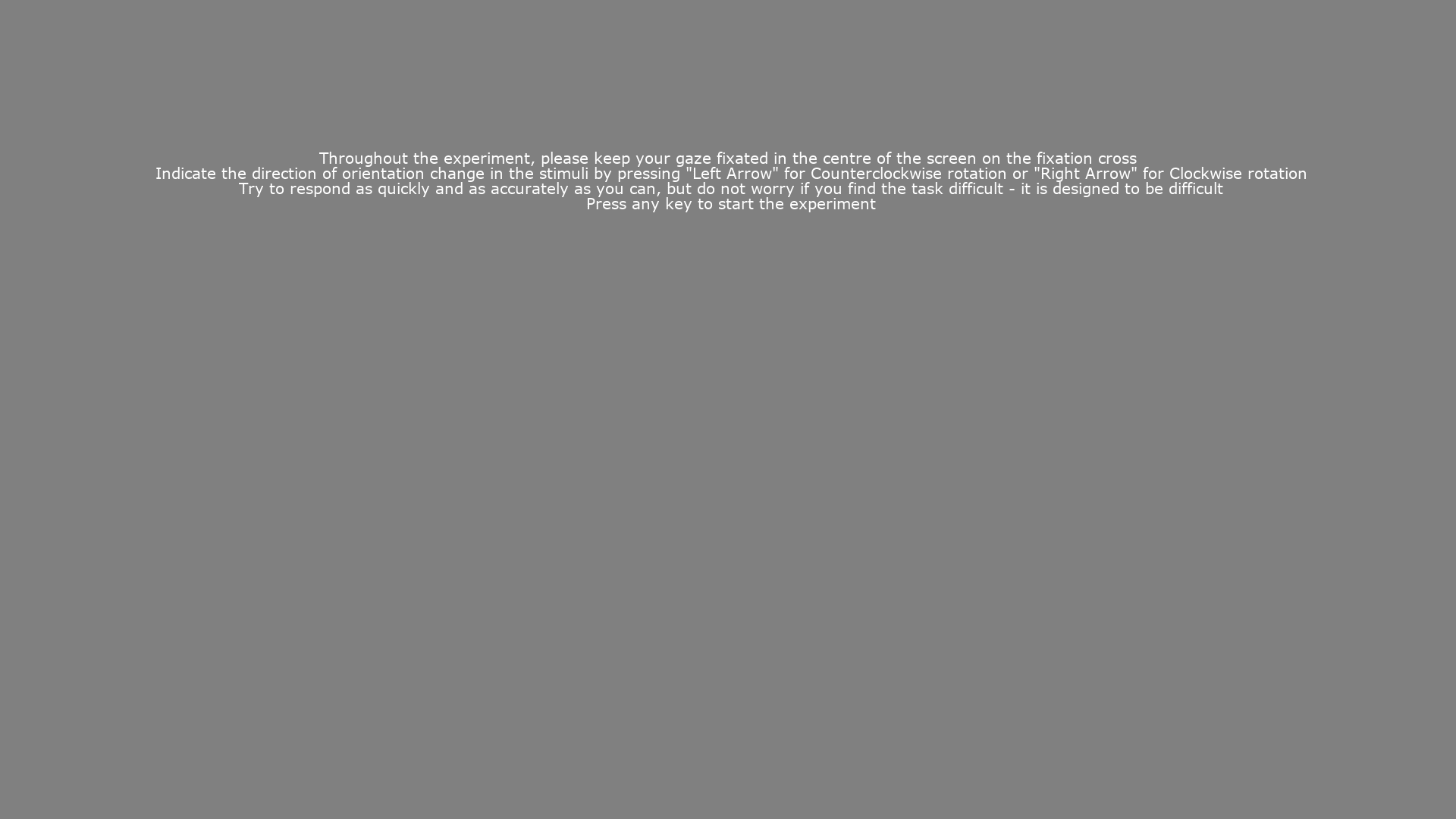

Supplement: Supplement 1 [file jovi-24-13-13_s001.zip › Experiment_2_Supplementary_Information/Experiment_2_Neutral_Cue_Type.gif]
